# Thorax, pelvis and hip pattern in the frontal plane during walking in unilateral transtibial amputees: biomechanical analysis

**DOI:** 10.1590/bjpt-rbf.2014.0032

**Published:** 2014

**Authors:** Francisco Molina-Rueda, Isabel M. Alguacil-Diego, Alicia Cuesta-Gómez, Javier Iglesias-Giménez, Andrés Martín-Vivaldi, Juan C. Miangolarra-Page

**Affiliations:** 1 Department of Physical Therapy, Occupational Therapy, Rehabilitation and Physical Medicine, Universidad Rey Juan Carlos, Alcorcón, Madrid, Spain; 2 Orthopedic Surgery and Traumatology Service, Hospital Universitario Virgen de las Nieves, Granada, Spain

**Keywords:** unilateral transtibial amputation, physical therapy, joint moments, frontal plane, kinematics, pelvis motion

## Abstract

**Background:**

Lower limb amputees exhibit postural control deficits during standing which can affect their walking ability.

**Objectives:**

The primary purpose of the present study was to analyze the thorax, pelvis, and hip kinematics and the hip internal moment in the frontal plane during gait in subjects with Unilateral Transtibial Amputation (UTA).

**Method:**

The participants included 25 people with UTA and 25 non-amputees as control subjects. Gait analysis was performed using the Vicon^(r)^ Motion System. We analyzed the motion of the thorax, pelvis, and hip (kinematics) as well as the hip internal moment in the frontal plane.

**Results:**

The second peak of the hip abductor moment was significantly lower on the prosthetic side than on the sound side (p=.01) and the control side (right: p=.01; left: p=.01). During middle stance, the opposite side of the pelvis was higher on the prosthetic side compared to the control side (right: p=.01: left: p=.01).

**Conclusions:**

The joint internal moment at the hip in the frontal plane was lower on the prosthetic side than on the sound side or the control side. Thorax and pelvis kinematics were altered during the stance phase on the prosthetic side, presumably because there are mechanisms which affect postural control during walking.

## Introduction

Lower limb amputation entails the loss of part of the motor system and affects the sensory system. For this reason, kinetics, kinematics, and the ability to walk are modified in people with lower limb amputations^1^ ^-^ ^3^. Human walking involves the coordination of limbs, pelvis, and thorax in all three planes^4^. Particularly, the motion in the frontal plane during walking contribute meaningfully to maintain postural control and moderate the total work during gait^2^ ^,^ ^3^. In this sense, during the stance period of the gait cycle, there is a large internal abductor moment at the hip that stabilizes the pelvis and, secondarily, the thorax^5^ ^,^ ^6^. The stabilization of the pelvis and thorax is essential to reduce the total work during steady-state walking. Therefore, an adequate motor pattern in the frontal plane helps to improve gait efficacy^7^ ^,^ ^8^.

To our knowledge, few studies have investigated thorax, pelvis, and hip kinematics and hip internal moment in the frontal plane in subjects with Unilateral Transtibial Amputation (UTA)^5^. Only one study analyzed differences in pelvis kinematics in the frontal plane in six men with transtibial amputations and three men with transfemoral amputations, compared to subjects without amputations^9^. Another study analyzed thorax and pelvis kinematics and hip internal moment in the frontal plane; however, they only studied the range of motion (ROM)^10^. Several studies have investigated the kinetic patterns of the joints of the lower extremities in the frontal plane in UTA during walking^11^ ^-^ ^13^, but none of those have analyzed the thorax and pelvis in that plane. Under these circumstances, it is relevant to consider particular events of thorax, pelvis, and hip kinematics during the gait cycle. In addition, the simultaneous analysis of kinematic parameters and internal moments in the frontal plane can elucidate the motor pattern that subjects with UTA employ in this plane during walking.

A detailed examination of the adaptations that occur due to unilateral transtibial amputation during gait in the frontal plane is essential to produce new physical therapy guidelines and new approaches which could improve the quality of life of these subjects and reduce their disability.

The primary purpose of the present study was to analyze thorax, pelvis, and hip kinematics and hip internal moment in the frontal plane during gait in subjects with UTA. It was hypothesized that subjects with UTA would demonstrate differences in thorax, pelvis, and hip kinematics and kinetics in the frontal plane on both the prosthetic and sound side compared to able-bodied individuals.

## Method

### Subjects

This study was approved by the Human Ethics Committee of Universidad Rey Juan Carlos, Alcorcón, Madrid, Spain (number 07/12), and informed consent was obtained from all of the participants. The participants included 25 individuals with UTA (23 men, 2 women; 12 traumatic, 10 vascular, and 3 tumoral) and 25 non-amputees as control subjects (21 men, 4 women). The unilateral transtibial amputees were recruited from several orthopedic clinics.

The control group matched the subjects with UTA in age, weight, and height. The inclusion criteria for the control subjects included walking independently without assistive devices and the absence of musculoskeletal and neurological disorders.

The subjects with UTA were wearing prostheses before being included. The minimum time since full adaptation to the prostheses was 6±9 months, on average. The post-amputation time prior to data collection was greater than or equal to 1 year (10.17±9.29 year, on average) for all persons with amputations. The prosthetic feet varied across subjects and included 19 energy storage and return (ESAR) prostheses and 6 single-axis feet prostheses. The socket types also varied across the subjects: 24 patients had total surface bearing (TSB) prostheses and 1 patient had a Kondylen Bettung Münster (KBM) prosthesis. All of the patients had either vacuum-assisted socket suspension or pin suspension, except for the patient with KBM, who had anatomical suspension. Prosthesis alignment and fit were verified by prosthetics expert. The subjects were tested in their original prostheses and alignment.

### Experimental protocol

**Figure 1:**
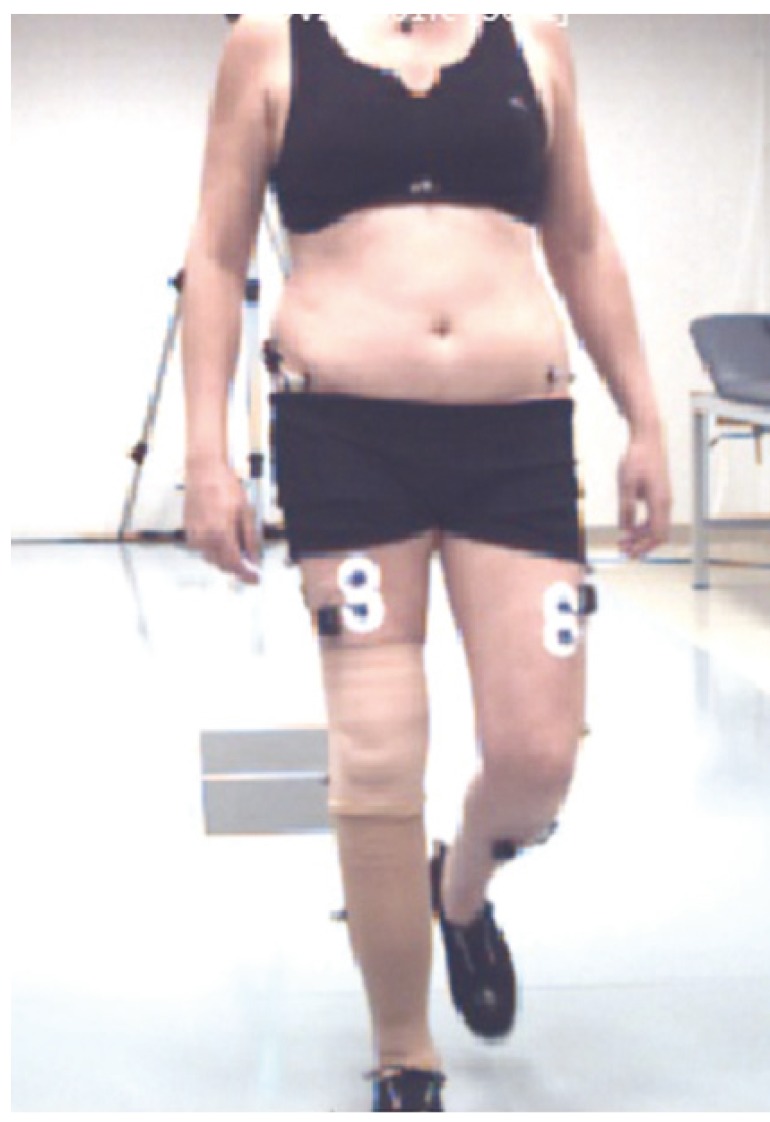
Special lightweight surface markers in volunteer with UTA.

Gait analysis was performed using the Vicon^(r)^ Motion System (Oxford Metrics, Oxford, UK). This system is a three-dimensional motion analysis system consisting of eight 100 Hz cameras with infrared strobes, two 1000 Hz AMTI^(r)^ force-plates (Watertown, USA), and a data station (Vicon MX control^(r))^ where the information was gathered and processed. Special lightweight surface markers (23) were attached directly to the skin or the prosthesis and placed over standardized landmarks on the sound side, prosthetic side, pelvis, and thorax or corresponding spots on the prosthesis [C7 vertebra, T10 vertebra, left and right acromion processes, right scapula, sternoclavicular joint (where the clavicle meets the sternum), sternum (xiphoid appendix), anterior and posterior superior iliac spines (left and right), lateral thigh, lateral femoral condyle, lateral shank, lateral malleoli, second metatarsal head, and posterior heel] according to the biomechanical model of the Vicon^(r)^ Plug-in Gait (Figure 1)^14^. On the prosthesis, the knee and ankle markers were attached to the spot corresponding to the lateral femoral condyle, lateral malleoli, second metatarsal head, and posterior heel on the sound side.

The Vicon^(r)^ Plug-in Gait is the next generation of the Vicon^(r)^ Clinical Manager software package. It employs the same model as referenced in the Vicon^(r)^ Clinical Manager with some additional features^14^. Tsushima et al.^15^ aimed to determine the test-retest reliability and inter-tester reliability of kinematic measures of the Vicon^(r)^ three-dimensional motion analysis system. Skin markers were placed on 15 defined pelvis and lower body locations in accordance with the Vicon^(r)^ Clinical Manager model. Coefficients of multiple correlation were calculated to evaluate the consistency between the kinematic variables across testers and sessions. Both test-retest and inter-tester reliability were high for motion in the frontal plane (pelvis obliquity=0.98; hip obliquity=0.97)^15^.

The subjects were instructed to walk along the 8-meter walkway while wearing their usual shoes (not athletic training shoes) and prosthesis. The participants were asked to walk at a self-selected comfortable gait speed.

### Data analysis

**Figure 2:**
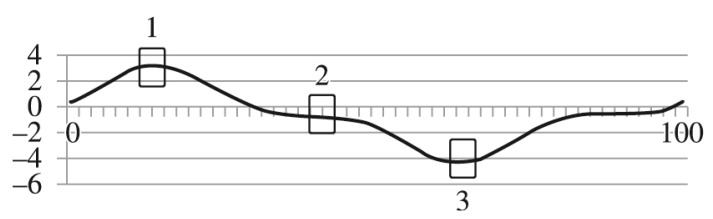
Pelvis motion in the frontal plane: peak pelvis obliquity. A positive pelvis obliquity value relates to a situation in which the opposite side of the pelvis is lower. A negative pelvis obliquity value relates to a situation in which the opposite side of the pelvis is higher. Peak pelvis obliquity: (1) Peak pelvis obliquity during loading response (0-10% GC); (2) peak pelvis obliquity during midstance (10-30% GC); (3) peak pelvis obliquity during pre-swing (50-60% GC). Y axis (degrees), X axis (0-100% gait cycle [GC]).

We analyzed the motion of the thorax, pelvis, and hip in the frontal plane. The following kinematic parameters were analyzed: peak value of thorax obliquity during the stance period; peak values of pelvis obliquity during the loading response, middle stance, and pre-swing phases (Figure 2); and the peak value of hip adduction during the stance period. Additionally, we analyzed the joint internal moment of the hip in the frontal plane, with special regard to the first peak of the hip abductor moment (middle stance) and the second peak of the hip abductor moment (terminal stance). Finally, we studied vertical ground reaction forces (GRFv) and spatio-temporal parameters, such as walking speed, cadence, and stride length.

The Vicon^(r)^ Nexus software v1.8.5 was used to calculate outcome measures based on the biomechanical model of the Vicon^(r)^ Plug-in Gait. The output angles for all joints were calculated from the YXZ cardan angles derived by comparing the relative orientations of the two segments. The pelvis and thorax markers were measured relative to the laboratory axes. The position of the hip segment was relative to the proximal segment, i.e. the hip to the pelvis. The course and direction of the segment axes are shown in the Vicon^(r)^ Plug-in Gait Product Guide-Foundation Notes Revision^14^. Joint moment calculations were determined from synchronized coordinate and force data using an inverse dynamics approach^7^. Joint kinetics was normalized to body weight, and all parameters were normalized to 100% of the gait cycle. Internal moments were calculated and interpreted as the forces developed by the muscle and ligaments counteracting the moments produced by the ground reaction force^16^.

Five gait cycles of the prosthetic side, the sound side, and the control side were averaged for the data analysis. The foot contact events were defined automatically, using the "autocorrelation events" option of the Vicon^(r)^ Nexus software v1.8.5.

### Statistical analysis

Statistical analysis was performed using SPSS 17.0. Shapiro and Wilk's W-statistic was used to screen all data for normality of distribution. The subjects were height/weight matched. Single-factor analysis of variance (ANOVA) and Bonferroni's adjustment a posteriori tests were used to compare the sound side, prosthetic side, right control side, and left control side. Walking speed comparisons between the controls and UTAs were determined using Student's t-test. A significance level of 0.05 was used for all statistical comparisons.

Thorax, pelvis, and hip kinematics and hip internal moment in the frontal plane were chosen as the aim outcome measures in this study. The effect size of these variables was estimated at 0.35. The alpha error was set to 0.05. The nonsphericity correction e was set to 1 with a statistical power of 0.9. It was estimated that 25 subjects would be required for each group (sound side, prosthetic side, and left or right control side) by using the software G*power 3.0.18^17^.

## Results

There were no differences between groups in age, height, weight or length of the lower extremities (Table 1). Healthy subjects and UTAs walked at a similar velocity, cadence, and with similar stride length (Table 2).

**Table 1:** Subjects characteristics.

	Subjects with UTA (n=25)		Control group (n=25)	
Age (years)	50.26 (13.76)		46.71 (14.79)	
Weight (Kg)	80.02 (13.79)		72.49 (9.73)	
Height (cm)	173.17 (8.55)		172.33 (8.36)	
	**Prosthetic side**	**Sound side**	**Right control side**	**Left control side**
Lower limb length (cm)	85.84 (4.81)	87.81 (5.32)	89.02 (5.25)	89.34 (6.02)

UTA : Unilateral Transtibial Amputation

Values are mean and standard deviation (SD).

**Table 2:** Spatio-temporal parameters. Kinematic data (degrees) of the hip, pelvis and thorax. Hip median peak values of internal moments in the frontal plane (Nm/Kg). Vertical ground reaction forces (GRFv, %BW).

Spatio-temporal parameters	Subjects with UTA (n=25)		Control group (n=25)	
	Prosthetic side	Sound side	Left control group (n=25)	Right control group (n=25)
Walking speed (m/s)	1.13 (.12)		1.20 (.14)	
Cadence (steps/min)	104.71 (7.67)		109.35 (6.78)	
Stride length (m)	1.29 (.18)	1.28 (.17)	1.22 (.14)	1.28 (.12)
** Parameters (frontal plane)**	**Prosthetic side**	**Sound side**	**Left control group (n=25)**	**Right control group (n=25)**
Thorax obliquity. Peak value during stance period.	–4.17 (2.95)*	–2.11 (3.54)	–.84 (2.47)	–.79 (2.21)
Pelvic obliquity Peak value during loading response.	1.73 (2.04)*	3.17 (2.44)	4.17 (2.29)	3.42 (2.41)
Pelvic obliquity. Peak value during middle stance.	– 2.16 (2.12)*	–1.26 (2.41)	.69 (2.34)	.38 (219)
Pelvic obliquity. Peak value during pre-swing phase.	**–3.01 (3.48)**	–1.38 (3.16)	–3.25 (2.63)	–4.12 (2.59)
Hip adduction. Peak value during stance period.	2.81 (3.35)*	5.05 (3.72)	5.44 (3.70)	5.67 (3.21)
First peak of the hip abductor moment	**.55 (.34)**	.77 (.23)	.73 (.15)	.70 (.22)
Second peak of the hip abductor moment	.72 (.24)*+	.93 (.36)	.89 (.14)	.82 (.09)
Fy1	**102.65 (13.76)+**	110.53 (9.51)	102.01 (7.57)	102.07 (8.01)
Fy2	97.75 (11.80)*+	105.57 (7.60)	111.60 (4.39)	111.62 (3.99)

Values are mean and standard deviation (SD)

* p<0.05 vs. Control side (right and left)

+ p<0.05 vs. Sound side

Fy1 . First peak of t he vertical GRF

Fy2 . Second peak of the vertical GRF

GRF . Ground Reaction Forces

UTA . Unilateral Transtibial Amputation.

**Figure 3:**
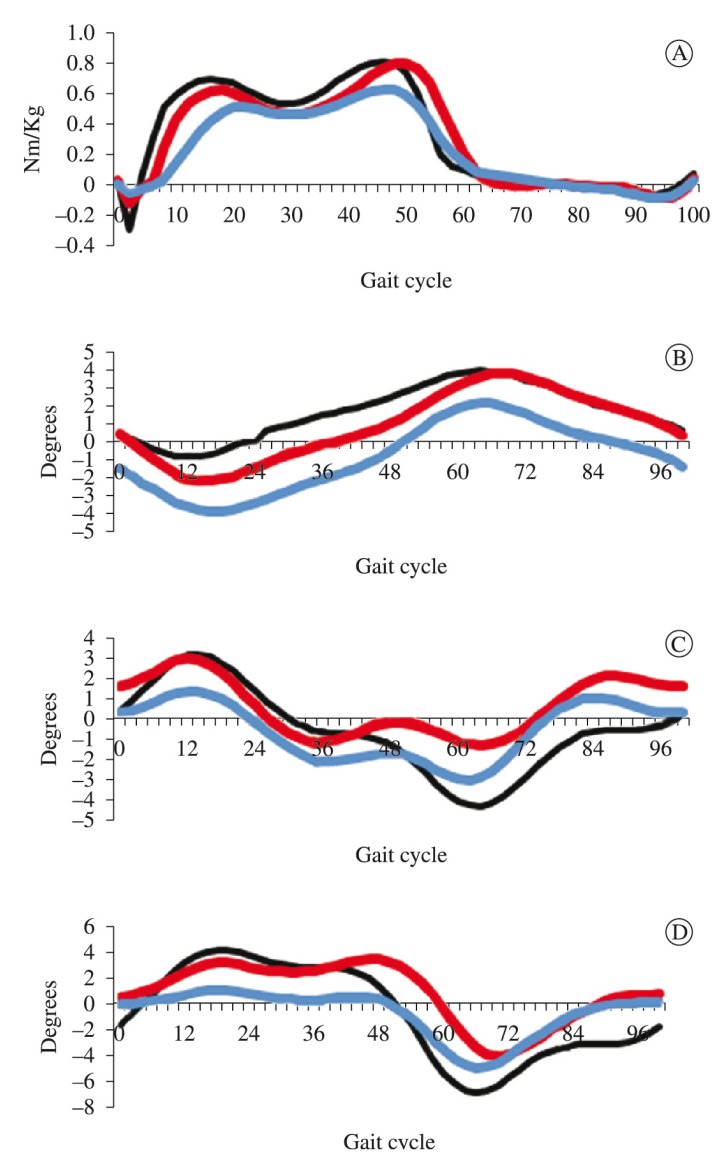
**(A) Hip abduction/adduction moment.** Positive values are abduction moment. Negative values are adduction moment. Y axis (Nm/Kg). X axis. (0-100% gait cycle). **(B) Mean values of thorax motion in the frontal plane.** A negative thorax obliquity angle relates to a situation in which the opposite side of the thorax is higher; a positive thorax obliquity angle relates to a situation in which the opposite side of the thorax is lower. Y axis (degrees). X axis. (0-100% gait cycle). **(C) Mean values of pelvis motion in the frontal plane.** A positive pelvis obliquity value relates to a situation in which the opposite side of the pelvis is lower. A negative pelvis obliquity value relates to a situation in which the opposite side of the pelvis is higher. Y axis (degrees). X axis. (0-100% gait cycle). **(D) Mean values of hip motion in the frontal plane.** Positive values are degrees of adducted position. Y axis (degrees). X axis. (0-100% gait cycle). Black line: Right control side; Blue line: Prosthetic side; Red line: Sound side.

Figure 3 highlights the kinematics of the thorax, pelvis, and hip and the hip internal moments in the frontal plane. The 4 graphs show the comparison between subjects with UTA and healthy subjects (control group). For the healthy subjects, we checked that the curves were the same for gait cycles on the right and left sides. Therefore, we chose to illustrate the mean curves obtained for the right side gait cycles.

### Joint internal moments and ground reaction forces

The first peak of the hip abductor moment showed no difference on the prosthetic side compared to the sound side (p=.13) and the control side (right: p=.06; left: p=.06). The second peak of the hip abductor moment was significantly lower on the prosthetic side than on the sound side (p=.01) and the control side (right: p=.01; left: p=.01; Table 2; Figure 3).

There were significant differences between the first and second peaks of GRFv generated by the prosthetic and sound sides (Table 2). The sound side produced significantly higher first and second peaks of GRFv than the prosthetic side (p=.04; p=.01).

### Joint kinematics

During normal gait, the unloading of the opposite limb removes the support for this lower limb, leading to a rapid pelvis and thorax drop just after loading response (positive peak value of pelvic obliquity during loading response). This movement is decelerated by the hip abductor muscles (hip abductor moment) of the lower limb that receive the load. A positive pelvic obliquity value relates to a situation in which the opposite side of the pelvis is lower. On the prosthetic side, the pelvis was closer to the neutral position compared to the control side (right: p=.01: left: p=.01) at the beginning of the stance.

Immediately after the loading response of the loaded lower limb, the pelvis and thorax of the opposite side rise during the stance period, reaching the maximum value in the pre-swing phase. A negative pelvic obliquity value relates to a situation in which the opposite side of the pelvis is higher. In our study, during middle stance, the opposite side of the pelvis was higher on the prosthetic side compared to the control side (right: p=.01: left: p= .01; Table 2; Figure 3).

In the frontal plane, a negative thorax obliquity angle relates to a situation in which the opposite side of the thorax is higher; a positive thorax obliquity angle relates to a situation in which the opposite side of the thorax is lower. On the prosthetic side, the thorax was higher than on the control side (right: p=.02; left: p=.01; Table 2; Figure 3).

During normal gait, the hip joint reaches peak adduction in the stance period. In our study, the peak hip adduction was lower compared to that of the control side (left: p=.04; right: p=.04).

## Discussion

In this study, the subjects with UTA walked with a reduced hip abductor moment during the stance phase. The hip joint was held in a slight adduction position compared to the sound side and the control side. Additionally, the subjects with UTA loaded their prosthetic side less than their sound side during natural cadence walking.

During normal walking, the load transfer over the stance side causes hip adduction of approximately 6-10º, controlled by the abductor muscles^5^. This pattern facilitates weight support during the loading response^5^ ^,^ ^6^. In this sense, several authors have shown that lower limb amputees walk with a reduced hip abductor moment^4^ ^,^ ^10^ ^-^ ^11^ ^,^ ^18^ and hip abducted position or with a slight hip abducted position^19^. This reduction in the load intensity of the prosthetic side and the kinematic and kinetic hip pattern observed in our study in the frontal plane during gait might be related to dysfunctional abductor muscles.

Alterations in lateral stability mechanisms can occur, either because the muscles are weak or because there are movements of the prosthetic side into the socket^20^ ^,^ ^21^. In any case, the internal abductor moment reduction observed in our study appears to affect thorax and pelvis kinematics. On the one hand, increased lateral thorax bending over the prosthetic side could reduce the lever arm and compensate for the dysfunctional abductors, as seen in transfemoral amputees or in patients with osteoarthritis^22^ ^,^ ^23^. On the other hand, the high position of the opposite side of the pelvis on the prosthetic side throughout the middle stance could be an indication of dysfunctional hip abductors^9^ ^,^ ^19^. A detailed examination of the activation patterns of the abductor muscles would provide additional insight into the motor pattern created by subjects with UTA.

Thorax and pelvis alignment in the frontal plane is influenced by the requirement to reach equilibrium around the hip joint, whereby the forces created by the lateral stability mechanism (abductor muscles) balance the loads imposed by body mass^24^. These forces create the abductor internal moment that is essential to support the weight of the body and maintain an upright posture during walking^5^ ^,^ ^10^ ^,^ ^11^. Therefore, the motion pattern observed in our study in the frontal plane might affect postural control during UTA gait and hinder the body's support on the prosthetic side. Several authors have found, in elderly subjects^25^ and in lower limb amputees^20^, a risk of falling with specific gait patterns on the sagittal plane. This relationship might also exist in the coronal plane, however this possibility should be investigated thoroughly.

### Study limitations

A potential limitation of this study was the model and the inverse dynamics technique, particularly in this context, in which there were both sound and prosthetic components. Furthermore, the placing of knee and ankle markers on the prosthesis at a location corresponding to the sound side might have affected the calculation of the joint centers. Additionally, the heterogeneous cohort and small sample size impeded the control of potential confounders, such as different times since amputation and since the current and first prosthesis prescription, differences in ages, differences in etiology of amputation and different prosthetic components. These aspects were not standardized across subjects. This conjuncture may affect our results as it will contribute to additional between-subject variance. Another limitation is the variability of the data. In our study, the standard deviation even in the control population was very high for some parameters.

## Conclusions

The conclusions that can be drawn concerning subjects with UTA, compared with healthy subjects, were as follows: (1) the joint internal moment at the hip in the frontal plane was lower in the prosthetic sides than in the sound sides or in non-amputees; (2) thorax and pelvis kinematics were alerted during the stance phase on the prosthetic side, presumably because there are mechanisms which affect postural control during walking.

Under these circumstances, the biomechanical pattern observed in our study in subjects with UTA in the frontal plane indicate that those need to receive specific physical therapy treatment, focusing to increase the proprioception and coordination of the proximal segments and abductor muscles.

## References

[1] Baraúna MA, Duarte F, Sanchez HM, Canto RST, Malusá S, Campelo-Silva CD (2006). Avaliação do equilíbrio estático em indivíduos amputados
de membros inferiores através da biofotogrametria computadorizada. Rev Bras Fisioter.

[2] Lamoth CJ, Ainsworth E, Polomski W, Houdijk H (2010). Variability and stability analysis of walking of
transfemoral amputees. Med Eng Phys.

[3] Van Velzen JM, Van Bennekom CAM, Polomski W, Slootman JR, Van der Woude LHV, Houdijk H (2006). Physical capacity and walking ability after lower limb
amputation: a systematic review. Clin Rehabil.

[4] Underwood AH, Tokuno CD, Eng JJ (2004). A comparasion of two prosthetic feet on the multi-joint
and multi-plane kinetic gait compensations in individuals with unilateral
trans-tibial amputation. Clin Biomech.

[5] Perry J (1992). Gait analysis: normal and pathological function.

[6] Inman VT, Ralston H, Todd F (1981). Human walking.

[7] Eng JJ, Winter DA (1995). Kinetic analysis of the lower limbs during walking: what
information can be gained from a three-dimensional model?. J Biomech.

[8] McKinnon CD, Winter DA (1993). Control of whole body balance in the frontal plane
during human walking. J Biomech.

[9] Michaud SB, Gard SA, Childress DS (2000). A preliminary investigation of pelvic obliquity patterns
during gait in persons with transtibial and transfemoral
amputation. J Rehabil Res Dev.

[10] Molina Rueda F, Alguacil Diego IM, Molero Sánchez A, Carratalá Tejada M, Rivas Montero FM, Miangolarra Page JC (2013). Knee and hip internal moments and upper-body kinematics
in the frontal plane in unilateral transtibial amputees. Gait Posture.

[11] Royer TD, Wasilewski CA (2006). Hip and knee frontal plane moments in persons with
unilateral, trans-tibial amputation. Gait Posture.

[12] Royer T, Koenig M (2005). Joint loading response and bone mineral density in
persons with unilateral, trans-tibial amputation. Clin Biomech.

[13] Fey NP, Neptune RR (2012). 3D intersegmental knee loading in below-knee amputees
across steady-state walking speeds. Clin Biomech.

[14] (2010). Vicon Plug-in Gait Product Guide-Foundation Notes Revision
2.0.

[15] Tsushima H, Morris HE, McGinley J (2003). Test-retest reliability and inter-tester reliability of
kinematic data from a three-dimensional gait analysis system. J Japan Phys Ther.

[16] Batteni H, Olney SJ (2002). Kinematic and kinetic variations of below-knee amputee
gait. J Prosthet Orthot.

[17] Faul F, Erdfelder E, Lang AG, Buchner A (2007). G*power 3: a flexible statistical power analysis program
for the social, behavioral, and biomedical sciences. Behav Res Methods.

[18] Sjödahl C, Jarnlo GB, Söderberg B, Persson BM (2003). Pelvic motion in trans-femoral amputees in the frontal
and transverse plane before and after special gait re-education. Prosthet Orthot Int.

[19] Vanicek N, Strike S, McNaughton L, Polman R (2009). Postural responses to dynamic perturbations in amputee
fallers versus nonfallers: a comparative study with able-bodied
subjects. Arch Phys Med Rehabil.

[20] Lilja M, Johansson T, Öberg T (1993). Movement of the tibial end in a PTB-prosthesis socket: a
sagittal x-ray study of the PTB-prosthesis. Prosthet Orthot Int.

[21] Convery P, Murray KD (2000). Ultrasound study of the motion of the residual femur
within a transfemoral socket during gait. Prosthet Orthot Int.

[22] Goujon-Pillet H, Sapin E, Fodé P, Lavaste F (2008). Three-dimensional motions of trunk and pelvis during
transfemoral amputee gait. Arch Phys Med Rehabil.

[23] Watelain E, Dujardin F, Babier F, Dubois D, Allard P (2001). Pelvic and lower limb compensatory actions of subjects
in an early stage of hip osteoarthritis. Arch Phys Med Rehabil.

[24] Grimaldi A (2011). Assessing lateral stability of the hip and
pelvis. Manual Ther.

[25] Hausdorff JM, Rios D, Edelberg HK (2001). Gait variability and fall risk in community-living older
adults: a 1-year prospective study. Arch Phys Med Rehabil.

